# Use of health communication materials by health-care providers for health education in low- and middle-income countries: A scoping review

**DOI:** 10.1371/journal.pone.0347576

**Published:** 2026-04-23

**Authors:** Amlaku Nigusie Yirsaw, Gebeyehu Lakew, Adane Nigusie

**Affiliations:** 1 Department of Health Promotion and Health Behavior, Institute of Public Health, College of medicine and health sciences, University of Gondar, Gondar, Ethiopia; 2 Health Research Development Directorate, Amhara Public Health Institute, Bahir Dar, Ethiopia; National Institute of Mental Health and Neurosciences: National Institute of Mental Health and Neuro Sciences, INDIA

## Abstract

**Background:**

Health communication materials (HCMs) are widely used to support health education and promotion activities in clinical and community settings. However, evidence regarding their use by healthcare providers in low- and middle-income countries (LMICs) remains inconsistent. This scoping review aims to systematically map and describe the existing literature on the utilization of health communication materials by healthcare providers in low- and middle-income countries (LMICs).

**Methods:**

This scoping review was conducted in accordance with the PRISMA-ScR reporting guideline and the Joanna Briggs Institute (JBI) framework for scoping reviews. The studies were searched from database inception to September 2025 using electronic databases (PubMed, ScienceDirect, Cochrane, and Hinari) were searched, and studies were selected based on predefined inclusion and exclusion criteria aligned with the Population–Concept–Context framework. Data extraction was performed using a standardized form, and findings were narratively synthesized spreadsheet by two independent reviewers. Findings were synthesized using descriptive numerical analysis and thematic analysis.

**Result:**

A total of 728 records were identified. 715 were screened after removing duplicates. Following title/abstract and full-text review, 18 studies were included. HCMs, including printed, audiovisual, and electronic tools, were covered in these studies. NGO support, perceived utility, and material accessibility were facilitators, while organizational limitations, patient disengagement, limited availability, and inadequate training were major obstacles.

**Conclusion:**

The use of health communication materials by healthcare providers in LMICs is influenced by contextual, institutional, and individual factors. While HCMs are widely available in many settings, their use remains inconsistent and is influenced by systemic challenges. Strengthening accessibility, provider training, culturally appropriate content, and integration into routine care may support improved implementation.

## Introduction

Low- and middle-income countries (LMICs) face the significant challenge of managing both communicable and non-communicable diseases simultaneously [1–3]. A substantial portion of this health burden stems from behavioral factors that cannot be fully addressed through medical interventions alone, emphasizing the critical need for effective health communication and education among healthcare providers at the individual, group, and community levels [4,5]. Health education is recognized as an essential element of primary health care and is pivotal in achieving the goal of “health for all.” It plays a crucial role in enhancing the quality of care at a reduced cost, promoting preventive behaviors, encouraging proactive health measures, and improving self-care practices [6–8].

Despite recognition of its importance, LMICs face barriers including limited access to well-designed educational materials, inadequate training for providers, and low health literacy among patients. Studies indicate that 40–80% of medical information provided is quickly forgotten [9], and a significant portion of what patients do remember is often inaccurate [10]. Health communication materials (HCMs) such as printed resources (posters, leaflets, flipcharts), audio tools (radio, mobile apps), and audiovisual aids (TV, videos, social media) enhance knowledge retention, reinforce key messages, and support behavior change [7,10,11]. However, current health education practices in LMICs often suffer from inadequate distribution, lack of culturally appropriate materials, and insufficient integration into routine healthcare services [12–15]. Although some studies show that HCMs can improve health literacy and patient outcomes, there is still limited evidence on how healthcare providers in LMICs actually use these materials in their daily practice. Most existing research focuses on patient outcomes or broad health promotion strategies, leaving an important knowledge gap regarding provider-level use.

Well-designed and accessible health communication materials may support patients’ understanding, help reduce misinformation, and have been reported to strengthen patient-provider interactions. For instance, research shows that giving patients written information after a consultation may increase how much they remember by up to 50% [14]. Consistent use of these materials in everyday practice may facilitate patient engagement, support preventive behaviors, and contribute to strengthening health systems, as reported in some studies [8,16].

This scoping review aims to systematically explore how healthcare providers in low- and middle-income countries (LMICs) use health communication materials (HCMs) for health education. The primary question focuses on utilization patterns, while secondary questions examine types of materials, health areas addressed, and barriers and enablers. there fore this scoping review study is exploratory and does not test causal hypotheses.

## Method

### Literature search and search methods

This scoping review aimed to identify and synthesize existing evidence to provide a comprehensive overview of the current knowledge on the use of health communication materials by Health-Care Providers for Health Education. The scoping review followed the PRISMA-ScR (Preferred Reporting Items for Systematic Reviews and Meta-Analyses Extension for Scoping Reviews) guidelines (S1 File), and adhered to the Joanna Briggs Institute (JBI) scoping review framework [17,18]. A thorough search was conducted across several electronic databases, including PubMed, ScienceDirect, Cochrane Library, and HINARI databases, covering both published and unpublished (grey) literature. Additionally, grey literature was searched using Google Scholar and general web searches through Google.

Searches were conducted from database inception to September 2025. Controlled vocabulary terms (e.g., MeSH terms in PubMed) and free-text keywords were combined using Boolean operators (“AND” and “OR”) to ensure comprehensive retrieval of relevant studies (S2 File). To further uncover relevant literature, references of included studies were manually searched, along with targeted searches on websites like the pubmed focused on health communication materials used by healthcare providers.

### Screening abstracts

Two independent reviewers screened titles and abstracts. Full texts of potentially eligible studies were assessed independently. Disagreements were resolved through discussion or consultation with a third reviewer.

### Scoping review research question

Using the Population–Concept–Context (PCC) framework recommended by the Joanna Briggs Institute, this review addressed the following:

Primary question:

- How are health communication materials used by healthcare providers to deliver health education in low- and middle-income countries?

Secondary questions:

- What types of health communication materials are utilized?
- In which health areas and service contexts are these materials implemented?
- What enablers influence their use?
- What barriers influence their use?

This review employed the PCC format to ensure that the study selection was aligned with these research questions. Eligibility of studies according to the participant, concept, and context (PCC) framework.

**Table:** 

Criteria	Elements
P: participants	Any health care providers giving health education
C: concept	Studies that used health communication material use for health education.
C: context	Low-and-Middle income countries

### Study selection criteria

Inclusion and exclusion criteria for the scoping review.

#### Inclusion criteria.

• Restricted to studies Published in English.
• Studies published Database inception to September 2025.
• Countries were classified as low- and middle-income according to the World Bank income classification at the time of study publication.
• This scoping review included observational (cross-sectional, case control, cohort and observational follow up), qualitative, and mixed-methods studies) that documented how healthcare providers in low- and middle-income countries used health communication materials for health education.

### Exclusion criteria

• Studies for which a full-text article could not be obtained (i.e., studies with no full-text were excluded after repeatedly contacting the authors) and non-primary research (e.g., review articles, conference and abstract)

### Operational definition

Health Communication Materials are defined as structured printed, audio, or audiovisual resources designed to provide health education to patients or communities. This definition encompasses printed materials (posters, leaflets, brochures, flipcharts), audio tools (radio programs, mobile messages), and audiovisual resources (videos, TV programs, digital apps). The operational definition of health communication materials was adapted from previous health communication literature [19,20].

Variables were extracted as reported in primary studies; no additional cut-offs or composite indices were imposed. We acknowledge that reliance on self-reported data may introduce recall bias and misclassification.

### Data extraction and management

A standardized spreadsheet was employed for data extraction, capturing study characteristics including title, author name, publication year, country, study method/design, participants, types of interventions, outcomes, and the health communication materials used. Two individuals (ANY and AN) independently extracted the data. The level of agreement between the reviewers was assessed using Cohen’s kappa coefficient [21]. The two authors resolved any disagreements through discussion, and if disagreements persisted, they consulted a third person (GL). Study characteristics, variable definitions, and measurement approaches were extracted exactly as reported in primary studies. All results are presented consistently in text, tables, and supplementary materials. This approach follows PRISMA-ScR guidelines to maximize transparency and reproducibility.

Data on variable definitions, measurement approaches, and categorization methods were extracted as reported in the original studies. This review did not impose additional classifications or cut-off values beyond those used by the primary studies.

### Data analysis and quality appraisal

Two complementary analytical approaches were used for presenting the findings [18]. First, a basic numerical analysis was conducted to assess the extent and distribution of the included studies. This analysis covered various aspects, including the geographical distribution of study settings, urban or rural contexts, types of publications, and the types of health education materials used, with the results displayed in tables and graphs. Second, the study findings from the existing literature were presented using thematic analysis. The narrative literature review was then organized around the themes derived from the study results. Themes were generated inductively from the extracted data and refined through iterative discussion among the review team [22–25].

Consistent with scoping review methodology, no inferential statistical analyses, subgroup analyses, or interaction tests were performed. Findings are summarized using descriptive statistics and thematic analysis. This approach ensures that all analyses are exploratory and aligned with the objectives of mapping evidence rather than testing hypotheses.

### Ethics approval and consent to participate

This section is not applicable because this study is a scoping review.

## Result

Database searching turned up a total of 728 records. 715 records were left for title and abstract review after 13 duplicate records were eliminated prior to screening. Following screening, 40 records remained for full-text evaluation, after 407 records were excluded based on title and abstract, and 268 were excluded because the full text was unavailable.twenty two of the 40 reports were disqualified since they did not fit the requirements for inclusion in the category of health

**Fig 1:**
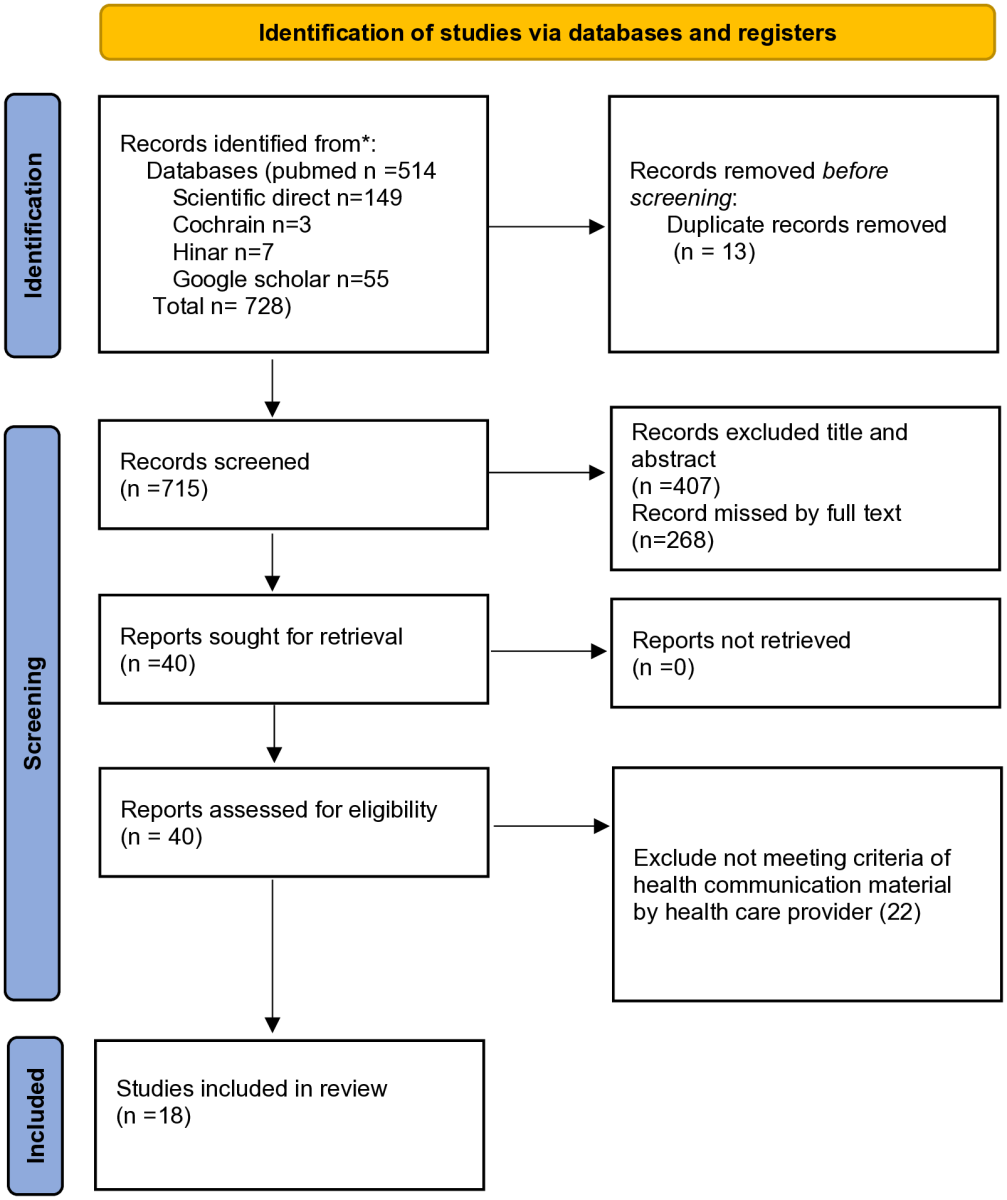
PRISMA flow diagram of the study selection process of use of Health Communication Materials by Health-Care providers for Health Education in low- and middle-income countries: a scoping review.

Communication materias used by healthcare professionals. Ultimately, this scoping review included 18 studies that met the inclusion criteria (Fig 1).

### Characteristics of included studies

A total of 18 studies met the inclusion criteria, encompassing 7 countries, with half conducted in Ethiopia. Study designs included observational (cross-sectional [8], case control (n = 1), cohort (n = 1) and observational follow up (n = 1)), mixed-methods (n = 6), and qualitative (n = 1) studies (Table 1) (Table 2).

**Table 1:** Articles about use of health communication material by country.

Article Setting by country	N	Percent (%)
Ethiopia	9	50
India	3	16.7
Nigeria	2	11.11
Namibia	1	5.56
Uganda	1	5.56
Iran	1	5.56
Ghana	1	5.56

**Table 2:** Characteristics of the included studies.

Author	Year of publication	Title	Study Design/method	Geographical focus	Types of health communication material use	Target out come	Key results	Limitation /recommendations
Birhanu et al	2011	Assessment of production and distribution of printed information education communication (iec) materials in ethiopia and utilization in the case of jimma zone, oromiya national regional state: a cross sectional study	Mixed	Ethiopia	printed Information Education Communication materials	To evaluate the process of producing, distributing, and utilizing printed Information Education Communication (IEC) materials and to identify current needs and gaps in IEC.	The IEC materials developed by the Health Education Extension Center and Oromiya Regional Health Bureau lacked cultural sensitivity and proper inventory management. A chronic shortage was reported, with only 68.0% of participants having used printed IEC materials. Of these, 48.2% found them highly understandable, but only 9.6% felt they considered the local context. Nurses and laboratory technologists used them less than environmental health experts, while private college graduates were 10 times more likely to use them than government institution graduates.	The study found that the design, production, distribution, and utilization of printed Information Education Communication (IEC) materials did not align with the fundamental principles of IEC material development. Therefore, all relevant institutions and individuals should work toward making improvements in these areas.
Kudzinesta	2020	Utility of medicines information leaflets in hypertensive care in a setting with low health literacy: A cross-sectional study	Mixed	Namibia	medicines information leaflets	To assess the level of health literacy and the effectiveness of medicine information leaflets (MILs) among hypertensive patients in public healthcare.	Among 139 patients, 63% were female, with a mean age of 45.7 years. Over 85.6% had low literacy skills, 38.8% needed help reading medicine information, and 66.9% struggled with health information comprehension. Access to and utilization of medicine information leaflets (MILs) were low (32.4% and 34.6%, respectively), mainly due to low health literacy, lack of guidelines, and non-native language MILs	To improve medication adherence among hypertensive patients in Namibia, health literacy programs and MIL guidelines are needed. Educational materials should include more visuals, be available in print and digital formats, and cover all aspects of CVD management. Follow-up studies can evaluate their effectiveness.
Thilaka	2017	Evaluation of Effectiveness of the Printed Educational Material (Brochure and Pamphlets) for Cardiovascular Diseases for Use among Patients	Mixed	India	Printed Educational Material (Brochure and Pamphlets)	To assess the effectiveness of the educational materials and to evaluate whether the population can effectively use these materials to understand and comprehend their content.	Sixty-four percent of respondents found the educational materials easy to understand, but the Apollo Hospital brochure was criticized for being text-heavy. While 56% liked its layout, only 20% found it comprehensive. Fifty-two percent suggested more visuals, and opinions on content adequacy were divided (48% sufficient vs. 44% inadequate). A majority (60%) preferred both print and online sources, emphasizing the need for better visuals and more comprehensive content for improved CVD patient education.	To improve educational materials, add visuals, engage in outreach, and assess effectiveness through follow-up studies. Offering information in print and digital formats and addressing content gaps will enhance patient support.
Gashu et, al	2021	Factors associated with women’s exposure to mass media for Health Care Information in Ethiopia.	Case-control	Ethiopia	mass media	To evaluate the factors influencing women’s exposure to mass media.	Factors affecting women’s media exposure include age 30–34 (AOR = 1.19), owning a mobile phone (AOR = 1.92), internet use (AOR = 1.56), living in a female-headed household (AOR = 0.76), and wealth index (middle: AOR = 1.48, richer: AOR = 2.13, richest: AOR = 2.67). Healthcare provider visits also influence exposure (AOR = 1.41).	Younger women, those in lower wealth quantiles, and those living in female-headed households were more likely to be less exposed to mass media. Enhancing household wealth and increasing access to information and communication technology (ICT) could boost women’s exposure to mass media.
Abita et, al	2022	Exposure to mass media family planning messages and associated factors among young men	Cross-sectional	Ethiopia	Mass media	To evaluate young men’s exposure to family planning messages and the factors influencing	The study found that most young men have limited exposure to family planning messages through mass media. Factors associated with higher exposure include educational level, owning a mobile phone, knowing where to obtain family planning methods, internet use, and viewing family planning as solely a women’s issue.	It is essential to improve the education level of youths and ensure they are informed about various channels for receiving family planning messages, making these resources easily accessible. Additionally, it’s important to shift the perception that family planning is solely a women’s issue and promote the idea that it requires cooperation from both parties.
Ewri, et, al	2018	Role of Mass Communication and Health Care In Promoting Glaucoma Awareness	Observational follow-up study	India	Use information-print media, electronic media (2 FM stations and one TV station	To assess the role of media and health agencies in promoting awareness and educating individuals about glaucoma.	Of the 26 hospitals studied, 77% were private and 23% were government-run. Only 7% of private hospitals had glaucoma awareness posters, and one hospital displayed a brochure. During Glaucoma Awareness Week 2016, a corporate eye hospital distributed pamphlets. While 61% of hospitals had TVs in waiting areas, none offered patient education programs. The average daily outpatient count per eye care facility was 109 ± 76, ranging from 35 to 275.	The study had limitations, including coverage of only two FM channels, exclusion of All India Radio, and no assessment of TV educational programs or social media usage. Glaucoma awareness was found to be limited, mostly during Glaucoma Awareness Week, emphasizing the need for ongoing awareness through various media and healthcare channels to prevent blindness.
Aderibigbe, et,al	2015	Barriers to health education in adolescents: health care providers’ perspectives compared to high school adolescents	Cross-sectional	Iran	School health education	To explore the barriers to health education as perceived by healthcare providers and compare these with the perspectives of adolescents.	The study revealed that from the perspective of healthcare providers, the major barrier to adolescents’ health education is the “lack of private rooms for educational sessions.” Conversely, adolescents themselves see a “lack of interest in the content of educational programs” as a significantly greater barrier to effective health education.	The results of this study provide valuable insights for shaping specific strategies in adolescent health promotion programs. Policymakers need to address both individual and contextual barriers affecting youth health education. Developing targeted strategies and policies to tackle these issues could help eliminate or mitigate these barriers. While it may take time for these strategies to show results, their benefits will ultimately be worthwhile.
Debele, et,al	2022	Health learning materials utilization for COVID-19 risk communication and community engagement among health workers	Mixed-methods	Ethiopia	Printed, audio, audio-visual	To evaluate the usage of COVID-19 health learning materials (HLMs) and identify the barriers and facilitating factors influencing their effectiveness.	The study found that 39.6% of respondents never used COVID-19 health learning materials (HLMs), 22.1% used them occasionally, and 38.3% used them regularly. Key factors influencing HLM utilization included the materials’ quality and usefulness, the working facility, educational level, and availability of HLMs, along with material-related, structural, and health worker-related factors.	To enhance health worker capacities, extensive training on health learning materials (HLMs) and risk communication should be provided at all levels. Policymakers and program implementers should revise national guidelines, including career structures for health education and promotion. Researchers are encouraged to focus on HLMs by investigating their production, quality, usefulness, and evaluating their implementation.
Bramo,et, al	2017	Utilization Status of Electronic Information Sources (EIS) for HIV/AIDS Care and Treatment in Specialized Teaching Hospitals of Ethiopia.	Mixed Method	Ethiopia	Electronic Information Sources (EIS)	To evaluate the utilization of Electronic Information Systems (EIS) for HIV/AIDS care and treatment in specialized teaching hospitals in Ethiopia	The study found that only 33.2% of health professionals used Electronic Information Systems (EIS) in their clinical practice for HIV/AIDS care. The main barriers were a lack of training (89.9%) and a preference for print resources (6.3%). Significant factors influencing EIS use included electronic information retrieval skills, the quality of information, and limited access to computers and the internet.	Hospital boards should develop strategies to enhance EIS utilization by fostering a culture of its use in clinical services. Additionally, priority should be given to improving the electronic information retrieval skills of health professionals and ensuring better access to computers and internet connections.
Medhanyie,et,al	2013	Meeting Community Health Worker Needs for Maternal Health Care Service Delivery Using Appropriate Mobile Technologies	Cohort	Ethiopia	Using Appropriate Mobile Technologies	To address the technical needs of Health Extension Workers (HEWs) and midwives in maternal health by utilizing appropriate mobile technology tools.	Most health workers quickly adapted to touchscreen devices with minimal technical support, and the unrestricted use of smartphones fostered a strong sense of ownership and empowerment. This motivation led to a low smartphone breakage rate of 8.3% and minimal loss at 2.7%. On average, health workers made 160 minutes of voice calls and downloaded 27MB of data per month, although SMS usage was low, with fewer than three messages sent per month.	Fostering a strong sense of ownership and empowerment among health workers is essential for the successful implementation of any mobile health program.
Arulchelvan, et, al	2016	Effective communication approaches in tuberculosis control: Health workers’ perceptions and experiences	Cross-sectional	India	Mobile phone	To examine health workers’ perceptions and experiences related to tuberculosis (TB) disease, patients, and effective communication strategies for TB control.	Health workers noted increased TB knowledge among DOTS patients, particularly about transmission and responsibilities. They highlighted the importance of regular patient interaction for adherence and found a media-mix strategy and mobile video player feature effective for raising awareness.	A combined approach of mass media and interpersonal communication is effective for TB control. Face-to-face interactions, patient-provider discussions, and TV-based content are particularly useful. Specialized materials should be developed for patients’ families, and smartphones can play a key role in implementing TB control programs effectively.
Chang, et, al	2011	Impact of a mHealth Intervention for Peer Health Workers on AIDS Care	Mixed	Uganda	Mobile phone	To assess the impact of public health workers (PHWs) on AIDS care.	29 PHWs at 10 clinics were randomized to receive an intervention involving phone communication with higher-level providers about patient-specific information. 970 patients were followed over 26 months, with no significant differences in virologic failure risk. Qualitative analyses revealed improvements in patient care and logistics, with broad support for the mHealth intervention. Challenges included inconsistent patient phone access, privacy concerns, and phone maintenance.	mHealth support intervention used by PHWs was successfully deployed in a rural, LMIC setting. While no significant quantitative impact was found in this exploratory study, qualitative and staff survey results demonstrated substantial benefits and support for the intervention. Further studies of mHealth tools used by health workers which focus on health systems and patient-oriented outcomes are warranted.
Andreatta, et, al	2011	Using cell phones to collect postpartum hemorrhage outcome data	Cross-sectional	Ghana	cell phones	To assess the use of cell phones by professional and traditional birth attendants in rural Africa for reporting postpartum hemorrhage (PPH) data.	During the 90 days following training, a total of 425 births and 13 cases of postpartum hemorrhage (3.1%) were reported. All attendants adhered to the reporting protocol, though the integrity of the data remained uncertain.	The results show that both professional and traditional birth attendants can be trained to use cell phones for real-time health outcome reporting. This approach provides a more accurate representation of events in remote communities. These findings could be applied to other program evaluations or population-monitoring efforts, particularly in rural areas, in healthcare and beyond
Geleta, et, al	2022	Investigating the Enablers and Barriers to Using Printed Information, Education, and Communication Materials Among Healthcare Providers	Qualitative	Ethiopia	Printed Information, Education, and Communication Materials	To explore the enablers and barriers to using printed Information, Education, and Communication (IEC) materials among healthcare providers	The study’s findings were categorized into four themes of enablers and four themes of barriers. The enablers included the availability of printed IEC materials, their distribution, perceived usefulness, and support from non-governmental organizations. The barriers were related to the printed IEC materials themselves, patient-related factors, healthcare provider-related factors, and government-related factors.	All relevant stakeholders should focus on enhancing the quality of IEC materials, providing training for healthcare providers, increasing the availability of these materials, and ensuring their distribution to health facilities.
Geleta, et, al	2022	Utilization of Printed Information, Education, and Communication (IEC) Materials and Associated Factors Among Healthcare Providers	cross-sectional	Ethiopia	Printed information, education, and communication materials	To evaluate the use of printed IEC materials and the factors associated with their utilization among healthcare providers	Of the 281 healthcare providers surveyed, 84% were aware of printed IEC materials, and 83.6% had used them, with 60.9% using them in the past month. Most participants (92.2%) planned to continue using them. Key factors influencing usage included age, sex, marital status, available time for health education, and recognizing the importance of IEC materials for basic concepts.	To boost the use of printed IEC materials, a coordinated effort from the federal government, regional authorities, non-governmental organizations, the zonal health bureau, and health facilities is essential.
Adanikin, et,al	2014	The Impact of Text Message Reminders on Increasing Postnatal Clinic Attendance	Cross-sectional	Nigeria	SMS	To test the hypothesis that SMS reminders would decrease non-attendance rates at postnatal clinics.	Out of 1,153 women in the intervention group, 1,126 (97.7%) received SMS reminders, resulting in a 21.3% failure-to-attend (FTA) rate, compared to 42.8% in the historic control group. This led to a 21.5% reduction in FTA rates, or 243 more women attending postnatal appointments. SMS reminders reduced non-attendance by 50% (relative risk 0.50; 95% CI, 0.32−0.77; P = 0.002). The cost for 2,252 SMS reminders over 6 months was 3,387 Naira (US $21.12), with an annual cost of US $42.24.	The study had limitations, including potential seasonal variations from the historic control group and uncertainty about whether text messages reached the correct recipients. Future research should explore two-way text messaging for better accuracy. Socio-demographic data was not available but likely had minimal impact. A randomized control trial would be valuable for further research, including prenatal appointments, child vaccinations, and drug compliance. In conclusion, SMS reminders reduced postnatal clinic non-attendance by 50%, suggesting they should be considered for implementation in maternal health services in Sub-Saharan Africa.
Ajiboye, et, el	2014	Knowledge and Use of Health Information and Communication Technologies (HICTs) Among Health Workers	Cross- sectional	Nigeria	health information and communication technologies	To investigate the use of health information and communication technologies (HICTs) by health workers across seven state hospitals and one private hospital.	The results indicate that while a significant number of respondents are aware of health information and communication technologies (HICTs), only a small proportion possess sufficient knowledge and actively use these technologies for healthcare delivery. Access to computers and relevant HICT resources is limited in the hospitals, with only one facility having an internet connection and none having a website.	There is a need for the management boards of hospitals and health workers to take advantage of the opportunities offered by health information and communication technologies (HICTs).
Handebo, et, al	2022	Using health communication materials to provide health education to professionals working in healthcare institutions.	Cross-sectional	Ethiopia	Printing, audio, and audio-visual	To evaluate the use of health communication materials among healthcare professionals working in health institutions	In a study of 298 healthcare professionals with a 96.4% response rate, 67.5% used health communication materials. The adjusted odds ratio (AOR) indicated that midwifery (AOR = 0.31) and laboratory professionals (AOR = 0.31), along with those not involved in health education (AOR = 0.09), were less likely to use them. Conversely, working at a health center (AOR = 3.60), having access to the materials (AOR = 2.23), and possessing good knowledge (AOR = 2.69) were positively associated with use.	The use of health communication materials by health professionals is essential for fostering positive health attitudes and behaviors. It is important to enhance the availability of these materials, coordinate health communication efforts, and strengthen the skills of health professionals.

The key insights drawn from these 18 articles are summarized and analyzed below.

### Themes in the studies

The review identified four primary themes that were commonly addressed: the types of HCMs used, the health areas targeted by these materials, and the barriers and enablers influencing their use.

#### 1. Use of health communication materials.

This scoping review highlights that while health communication materials, including printed items like posters, leaflets, and flipcharts, as well as audio and audiovisual tools such as television, radio, mobile apps, and YouTube videos, are available, they are not widely utilized by healthcare providers in low- and middle-income countries.

#### 2. Health areas addressed by health communication materials.

In this scoping review, the health communication materials and healthcare provider interventions in low- and middle-income countries specifically address a range of critical health areas. These include hypertension, tuberculosis (TB), human immunodeficiency virus (HIV)/acquired imsmunodeficiency syndrome (AIDS), child health, maternal health service delivery, cardiovascular disease, and glaucoma prevention. Additionally, they focus on hemorrhage prevention and treatment, risk communication and community engagement during COVID-19, and family planning service utilization. These efforts also encompass broader health campaigns and related topics, all aimed at improving public awareness, prevention, and management of these health conditions. Such initiatives are vital for promoting overall public health and ensuring better health outcomes for communities.

#### 3. Enablers of the use of HCMs by health care providers.

**3.1 Availability of HCMs.** Some Health facilities in low- and middle-income countries commonly have a variety of HCMs available. These include printed educational materials such as posters, flipcharts, brochures, leaflets, cards, and charts, as well as audio resources like phone-based messages, mass media (TV), and other electronic devices like the internet.

**3.2 Culture and programs of distribution and use of HCMs.** There is a well-established practice of distributing printed HCMs both within and outside health facilities to convey health-related messages. Posters and flipcharts on topics like antenatal care, EPI, TB, and HIV prevention and control are regularly utilized to enhance health education. Additionally, health issues are frequently addressed on TV channels, with scheduled programs that focus on national health priorities, where health experts are assigned to discuss these critical topics.

**3.3 Perceived usefulness of HCMs by health care providers.** The findings indicated that healthcare providers’ perceptions significantly impact the utilization of health learning materials. Most respondents emphasized that these materials are crucial for delivering health information, enhancing health service utilization, reducing medical errors, serving as guidelines, and accessing updated information.

**3.4 Support from a Non-Government Organization.** The use of printed HCMs, as well as audio and audiovisual educational resources, was significantly boosted by strong support from non-governmental organizations.

#### 4. Barriers to the use of HCMs by healthcare providers.

**4.1 HCMs related barriers.** The studies reviewed highlighted several barriers related to health communication materials, including language barriers, the lack of appropriate health learning materials, and challenges with accessibility and availability, all of which significantly hinder their use.

4.2 Patient-related barriers. A lack of interest among patients in engaging with HCMs was identified as a major barrier.

4.3 Healthcare provider-related Barriers. Barriers related to healthcare providers included work overload, insufficient knowledge and skills, inadequate training in the use of health communication materials, and negative attitudes toward these materials.

4.4 Government-related barriers. A lack of government attention and concern for health education communication materials was identified as a significant barrier.

## Discussion

This scoping review found that several included studies reported positive outcomes associated with the use of health communication materials, including improved service delivery and patient engagement. However, due to the predominance of descriptive and heterogeneous study designs, causal conclusions regarding effectiveness cannot be drawn. HCMs have been described as playing an important role in maternal health, as well as in the prevention and treatment of communicable and non-communicable diseases. The successful use of these materials is often influenced by contextual and systemic factors rather than proven intervention effects. Key factors such as the development of evidence-based health policies, access to reliable internet, mobile networks, and electricity, and the production and distribution of printed materials were reported in some studies as potentially relevant, but these observations are hypotheses and not confirmed causal mechanisms [26,27]. As a scoping review, this study maps the available evidence and identifies knowledge gaps rather than evaluating intervention effectiveness.

Although several included studies reported positive outcomes related to the use of health communication materials, these findings should be interpreted with caution. Most of the included studies were cross-sectional in design, which means they can only show associations, not cause-and-effect relationships. Therefore, any suggested mechanisms or potential benefits should be viewed as preliminary insights that warrant further investigation, rather than as confirmed effects

The review suggests that the use of HCMs by healthcare providers in LMICs for addressing health issues is relatively low [28–30], although it has been gradually increasing. The literature reviewed spans from database inception to September 2025, with a significant concentration of studies published between 2020 and 2022, likely driven by the COVID-19 pandemic and ongoing challenges like TB, TB/HIV co-infection, and HIV/AIDS in LMICs. Moreover, there has been a noticeable rise in studies addressing non-communicable chronic diseases. These trends describe the existing evidence but do not imply causal effects of HCMs on outcomes.

The patterns found in our LMIC-focused review appear to be universal when compared to other global reviews. For example, evaluations of mHealth behavior change communication interventions in developing nations have revealed conflicting data regarding the efficacy of these interventions, with numerous studies exhibiting inconsistent effects on behavior change outcomes and lacking thorough evaluation. When studies differ greatly in terms of design and methodological rigor, it can be difficult to determine the efficacy of health communication materials [31].

These findings can be further interpreted by incorporating well-established theoretical frameworks of behavior modification and health communication. Perceived susceptibility, perceived severity, perceived benefits, perceived barriers, and self-efficacy are factors that influence people’s engagement with health information and subsequent behavior change, according to the Health Belief Model (HBM), which is widely used to design and understand health behavior interventions. This can help explain why patients frequently prioritize receiving immediate clinical care over educational materials [32]. Adistionally The Diffusion of Innovations Theory posits that adoption of new tools depends on perceived advantage, compatibility, complexity, trialability, and observability, which may explain limited use of HCMs when materials do not fit existing workflows or lack support [33].

Most of the research has focused on African and Asian countries, and while the presence of research in this field is growing, much of it has been non-experimental and descriptive. Notably, Ethiopia was the site of a sizable percentage of the included studies. Rather than selection bias, this concentration represents the current distribution of published evidence. Given that half of the included studies were conducted in Ethiopia, findings may disproportionately reflect health system and policy contexts specific to that setting. The greater amount of research in this field may be partially explained by Ethiopia’s robust health extension programs and extensive primary healthcare system, which actively incorporate health communication techniques. Although Ethiopia and many other low-income nations have structural and resource-related similarities, the implementation and use of HCMs may be impacted by contextual variations in the infrastructure, cultural norms, and policy environments of the various LMICs.

HCMs in LMICs are predominantly produced by governmental and non-governmental organizations, targeting all populations, particularly high-risk communities. These materials have been especially useful in managing outbreaks, crises, disasters, and the prevention of communicable and non-communicable diseases.

Although the evidence base on the use of health communication materials is expanding, it remains somewhat limited. The most commonly studied outcomes include increased uptake of maternal health services, such as family planning, post-partum hemorrhage prevention and treatment, antenatal care (ANC), postnatal care (PNC), and facility-based deliveries. Several studies reported favorable outcomes associated with HCM use, although variability in methodological rigor limits definitive conclusions regarding effectiveness [34–37].

This review summarizes reported outcomes related to infectious diseases such as HIV/AIDS and TB; however, the strength of evidence varies across studies [38–40]. While further research is needed to strengthen the evidence base for other health-related outcomes, there should be an increased focus on evaluating the effectiveness of interventions that address multiple health practices [41,42].

The review identifies several barriers to the accessibility and availability of health communication materials, including inadequate infrastructure, insufficient funding, logistical challenges in distribution, and a lack of training for healthcare professionals on using these materials. These obstacles contribute to the limited use of health communication strategies in healthcare settings [27,43,44]. Previous studies have shown that healthcare providers often deprioritize behavior change communication due to a lack of refresher training and the need to focus on acute diseases. Similarly, studies conducted in Texas found that heavy job burdens were the main reason for inadequate health promotion practices [45].

Language barriers and the lack of suitable materials also contribute to the underutilization of these resources. This suggests that the absence of appropriately tailored materials for various outpatient departments (OPDs) and wards, along with the lack of printed materials in local languages, are key factors limiting their use by healthcare professionals. These findings align with studies conducted in other regions [46–48]. An additional barrier identified is the lack of patient interest in engaging with printed, audio, and audiovisual materials. The findings indicate that clients visiting health facilities are primarily focused on receiving medical care and show little interest in health education or health-related television programs. This lack of interest is consistent with findings from previous studies [45,49]. Comparative global evidence suggests that multifaceted communication strategies that combine community engagement, tailored messaging, and supportive systems tend to be more effective than single‑channel approaches, emphasizing the need for integrated communication frameworks in future health education interventions globally.

### Implications of this scoping review

The findings of this scoping review offer important insights for healthcare organizations, professionals, researchers, and the general public interested in enhancing the use of HCMs in LMICs. Although the existing body of literature on this topic is somewhat limited. however, most of the studies are descriptive in nature. HCMs can contribute to improved public health by supporting disease surveillance, mass communication, health education, knowledge translation, and collaboration among healthcare providers. However, concerns about the quality of information and the risk of misinformation present challenges to their use. Poorly communicated or incorrect information could lead to harmful health behaviors and negative outcomes for consumers. To address these challenges, it is crucial to promote the use of credible HCMs and to educate the public on the responsible use of social media to minimize the spread of misinformation. Furthermore, the rapid advancements observed in the use of HCMs in LMICs could provide valuable lessons, potentially leading to similar progress in other health-related areas or sectors such as education.

## Limitations

This review has several limitations. We included only studies published in English, which may have excluded relevant evidence in other languages. Focusing solely on LMICs limits the applicability of findings to high-income settings.

The included studies varied widely in design, quality, and outcome measurement, limiting direct comparability and introducing residual and unmeasured confounding. Factors such as provider experience, patient socio-economic status, facility resources, or health literacy may have influenced outcomes but were not consistently reported or controlled for. Additionally, many studies relied on self-reported data, which may be affected by recall bias, social desirability bias, and inconsistencies in measurement across settings, leading to potential information bias.

While we examined both printed and digital materials, differences in access to technology such as internet connectivity, mobile phone availability, and electricity could not be fully accounted for publication bias is possible, as negative or inconclusive studies may not have been published or captured, despite searching grey literature. Excluding review articles to avoid duplication may have limited our ability to capture broader implementation barriers often reported in systematic reviews.

Most studies were descriptive and non-experimental, restricting causal inference. As is common in scoping reviews, no formal quality appraisal was conducted, limiting the ability to judge evidence strength and calling for cautious interpretation.

Some primary studies may not have fully accounted for potential confounding factors, which could influence the observed associations. Another important limitation is that a large proportion of the included studies were conducted in Ethiopia, which may limit the applicability of the findings to other low- and middle-income countries. Differences in healthcare systems, provider training, resource availability, and health literacy across settings may affect how health communication materials are used and implemented.

The efficacy of HCMs cannot be fully interpreted because this scoping review did not include a formal critical appraisal of the studies. Rather than assessing study quality, the goal was to map and compile the available evidence. The results may therefore be influenced by differences in study design, reporting, and methodological rigor. Future systematic reviews that include quality appraisal are needed to provide stronger evidence.

The findings of this review should also be considered in light of differences in definitions and measurement approaches across studies, which made direct comparison more difficult.

## Conclusion

This scoping review mapped the current evidence on the use of HCMs by healthcare providers in LMICs. The results show that while different types of materials are accessible and utilized across multiple health areas, their use is influenced by contextual and systemic challenges. Barriers include limited access, inadequate training, language constraints, and organizational factors. Although some studies report positive outcomes associated with HCM use, this review does not establish causal effectiveness. Strengthening implementation strategies, improving accessibility, and investing in provider training may enhance the integration of HCMs into routine healthcare delivery.

## Recommendation

### Short-term (Facility-level)

- Ensure availability and accessibility of HCMs (printed, audio, and audiovisual) at points of care.
- Integrate interactive elements into HCM use during patient consultations to improve engagement.
- Promote the use HCMs within health facilities to support timely access to health education materials.

### Medium-term (Training & guideline updates)

- Provide comprehensive training for healthcare providers on HCM use, and risk communication strategies.
- Update national and facility-level guidelines to integrate standardized health communication practices.
- Enhance cultural relevance and context-specific adaptation of materials to better meet patient needs.

### Long-term (Policy & Systems Strengthening)

- Strengthen national policies and career structures to support health education and promotion.
- Foster coordinated collaboration between federal/regional authorities, NGOs, and health facilities for the production, distribution, and monitoring of HCMs materials.
- Invest in sustainable health communication systems, including digital infrastructure, consistent funding, and evaluation mechanisms to monitor effectiveness and impact.

## Supporting information

S1 File PRISMA-ScR checklist. (PDF)

S2 File Searching stratagey of included studies. (DOCX)
