# Supplementary material for: Use of health communication materials by health-care providers for health education in low- and middle-income countries: A scoping review
Source: PLoS One. 2026 Apr 23;21(4):e0347576. doi: 10.1371/journal.pone.0347576 (PMC13105348; doi:10.1371/journal.pone.0347576)
Supplement: S2 File — (DOCX) [file pone.0347576.s002.docx]

**S2. Searching strategy of included study**

| **Database** | **Filters / Specified By** | **Date of Search** | **Full Search String** | **Number of Articles Retrieved** |
| --- | --- | --- | --- | --- |
| **ScienceDirect** | Title; Article type = Research article; Language = English | September 2025 | ("Use of Health Communication Materials by Health-Care Providers for Health Education in Low- and Middle-Income Countries: A Scoping Review") | 149 |
| **PubMed** | Full text; Article type = Clinical Trial Protocol, Clinical Trial Phase I–IV, Randomized Controlled Trial; Language = English | September 2025 | ("Health Communication" AND "Materials" AND "Use" AND "health education") OR "health promotion" AND ("health care provider" OR "health worker") AND "Low and Middle-Income Countries" | 514 |
| **Cochrane** | Full text; MeSH terms; Language = English | September 2025 | (Health communication materials AND Health education AND (Health care provider* OR health professional*) AND (Lower Middle-Income Countries* OR developing countries)) OR (Health communication materials AND (Health education* OR health information* OR health promotion*) AND (Health care provider* OR health worker*) AND Lower Middle-Income Countries) | 3 |
| **Hinari** | Full text; Language = English | September 2025 | " Use of Health Communication Materials by Health-Care Providers for Health Education in Low- and Middle-Income Countries: A Scoping Review" | 7 |
| **Google Scholar** | Full text; Language = English | September 2025 | "Health Communication Materials" AND "Use" AND ("health education" OR "Health promotion" OR "Health information") AND ("health care providers" OR "health care worker") | 55 |
